# A functional neuroimaging study of fusiform response to restricted interests in children and adolescents with autism spectrum disorder

**DOI:** 10.1186/s11689-016-9149-6

**Published:** 2016-04-14

**Authors:** Jennifer H. Foss-Feig, Rankin W. McGugin, Isabel Gauthier, Lisa E. Mash, Pamela Ventola, Carissa J. Cascio

**Affiliations:** Yale University Child Study Center, 230 South Frontage Rd, New Haven, CT USA; Seaver Autism Center for Research and Treatment, Department of Psychiatry, Icahn School of Medicine at Mount Sinai, One Gustave Levy Place Box 1230, New York, NY USA; Department of Psychology, Vanderbilt University, 2301 Vanderbilt Place, Nashville, TN USA; Department of Psychiatry, Vanderbilt University, 1601 23rd Ave South, Suite 3057, Nashville, TN 37212 USA; Vanderbilt Kennedy Center, Nashville, TN USA

**Keywords:** Autism spectrum disorder, Fusiform face area, Restricted interests, Expertise, fMRI, Fusiform gyrus

## Abstract

**Background:**

While autism spectrum disorder (ASD) is characterized by both social communication deficits and restricted and repetitive patterns of behavior and interest, literature examining possible neural bases of the latter class of symptoms is limited. The fusiform face area (FFA) is a region in the ventral temporal cortex that not only shows preferential responsiveness to faces but also responds to non-face objects of visual expertise. Because restricted interests in ASD are accompanied by high levels of visual expertise, the objective of this study was to determine the extent to which this region responds to images related to restricted interests in individuals with ASD, compared to individuals without ASD who have a strong hobby or interest.

**Methods:**

Children and adolescents with and without ASD with hobbies or interests that consumed a pre-determined minimum amount of time were identified, and the intensity, frequency, and degree of interference of these interests were quantified. Each participant underwent functional magnetic resonance imaging (fMRI) while viewing images related to their personal restricted interests (in the ASD group) or strong interest or hobby (in the comparison group). A generalized linear model was used to compare the intensity and spatial extent of fusiform gyrus response between groups, controlling for the appearance of faces in the stimuli.

**Results:**

Images related to interests and expertise elicited response in FFA in both ASD and typically developing individuals, but this response was more robust in ASD.

**Conclusions:**

These findings add neurobiological support to behavioral observations that restricted interests are associated with enhanced visual expertise in ASD, above and beyond what would be expected for simply a strong interest. Further, the results suggest that brain regions associated with social functioning may not be inherently less responsive in ASD, but rather may be recruited by different environmental stimuli. This study contributes to our understanding of the neural basis of restricted interests in ASD and may provide clues toward developing novel interventions.

**Electronic supplementary material:**

The online version of this article (doi:10.1186/s11689-016-9149-6) contains supplementary material, which is available to authorized users.

## Background

Autism spectrum disorder (ASD) is associated with deficits in social abilities and interest, often combined with restricted patterns of behavior that include increased attention to and focus on narrow topics of interest [1]. A majority of neuroimaging studies in ASD have focused on abnormalities in the social brain [44], which have been associated with behavioral deficits including atypical eye contact, reduced attention to faces, and poor theory of mind [13, 41, 42]. Functional abnormalities have been identified in several brain structures, including the amygdala, superior temporal sulcus (STS), and a functionally-defined, face-selective area of the fusiform gyrus commonly referred to as the fusiform face area (FFA; [25, 26]). While these studies have explored the role of the social brain in ASD symptoms, the literature examining possible neural bases of increased levels of repetitive behaviors and circumscribed interests is far more limited.

Over the past two decades, a growing body of research has indicated that children with ASD show increased attention to and preference for non-social stimuli at the cost of reduced interest in social stimuli (e.g., faces), which are generally preferred by typically developing children [7, 31, 40]. Klin and colleagues [29, 30] have suggested that individuals with autism find objects, rather than social stimuli, to be most salient. This atypical attentional bias may lead to increased interaction with and preference for the non-social environment at the expense of social development. Infants and young children with ASD show greater preference for viewing objects relative to faces [35, 50], spurring suggestions that atypical attentional biases early in development might initiate a cascade of social deficits observed in ASD across the lifespan [55]. Indeed, topics of circumscribed interest elicit greater positive affect and arousal than do social stimuli in older individuals with ASD [48].

A relatively new line of research suggests that the neural reward system is differentially responsive in individuals with ASD, which may explain the atypical preferences individuals with ASD display for non-social stimuli [9, 10]. For example, whereas social stimuli (i.e., faces) activate neural reward circuitry in typical individuals [32], this is not the case for individuals with ASD [14, 58]. However, these differences do not seem to reflect pervasive deficits in reward system responsivity in ASD. Intact or enhanced responses in reward regions have been shown in response to monetary reward [12, 39, 54], object images as rewards in gambling paradigms [15], and food images under mild fasting conditions [5]. Most recently, individuals with ASD were found to show *increased* reward system response relative to controls in response to images of their particular areas of interest [4]. It remains unknown to what extent images related to restricted interests may engage other neural regions that are associated with processing social stimuli. Moreover, whether social motivation, expertise, and skill deficits are primarily related to altered reward system tuning versus broader neural differences, such as in visual processing, is a topic of debate.

The fusiform gyrus, and the FFA in particular, has consistently been found to respond atypically in ASD. The FFA is a region in the ventral temporal cortex that shows preferential responsiveness to faces in typical individuals [25, 26] and is thought to respond selectively to static, invariant aspects of human faces, such as identity. This function is in contrast to other regions of the social brain, such as the posterior STS, which respond to dynamic aspects of faces, such as expression [2]. However, the FFA is not exclusively responsive to faces, as it also responds preferentially when observers identify non-face objects from domains for which they have high levels of visual expertise [19]. According to the expertise hypothesis, in typical individuals, faces represent merely one example of a stimulus class for which people have developed significant identification expertise [61]. Thus, though the FFA is an integral part of the social brain, it is also involved in the processing of other, often non-social, stimuli for which individuals have visual expertise. Individuals with ASD show diminished FFA response to face stimuli [8, 53, 56], though there is evidence that their FFA responds more typically when viewing pictures of familiar faces [45], animal faces [67], or when visual scan paths of faces are directly manipulated [43]. Additionally, individuals with milder autism symptoms may show relatively intact FFA response to faces [52]. There is very little information, however, about how the fusiform gyrus responds to non-face objects of perceptual expertise in individuals with ASD. Given the expertise associated with highly restricted or circumscribed interests, it is of interest to determine whether images related to these interests recruit the FFA in ASD.

The lack of cortical face specialization in ASD may be a byproduct of broadly reduced social interest, leading to reduced attention to and regard for faces and thus reduced expertise for faces [11, 20]. Interestingly, a single-subject study examining FFA function in a boy with ASD and a strong interest in Digimon demonstrated that presenting images of Digimon characters reliably engaged fusiform regions where human face images did not [21]. The ability of fictional characters for which children have intense interest, and presumably great expertise, to elicit robust FFA activation has also been demonstrated in typically developing children [22]. Combined, these studies support the notion that, beyond its role as part of the social brain, the FFA is preferentially responsive to topics of intense interest in both typically and atypically developing children. Grelotti and colleagues suggested that differential engagement of FFA may have been the result of differences in the relative amount of time the individual with ASD spent looking at or thinking about people versus Digimon over his lifetime, resulting in differing levels of expertise and cortical specialization [21]. More broadly, their finding suggests that FFA can indeed respond preferentially to highly familiar classes of stimuli in ASD. However, this case study has yet to be replicated in a larger sample of individuals with ASD. In addition, it remains unknown whether objects of restricted interest and expertise that do not consistently include faces (as Digimon characters do) will also engage the FFA in individuals with ASD.

In the present study, we examined response of the fusiform gyrus to individualized stimulus sets reflecting images of participants’ particular areas of interest in a sample of children and adolescents with and without ASD. In this same sample, individuals with ASD had increased reward system response to images of their intense interest [4]. Here, we extended our examination of atypical neural specialization and the neural basis of restricted interests in ASD to the fusiform gyrus. In keeping with the assumption that individuals with ASD often develop high levels of perceptual expertise for objects or scenes related to their restricted interests, we hypothesized that children and adolescents with ASD would show enhanced activation of fusiform regions in response to these images relative to individuals with typical development who had a strong but less intense or all-consuming hobby or interest.

## Methods

### Participants

We tested 21 male children and adolescents with a diagnosis of ASD and 23 typically developing (TD) male controls group-matched for age and IQ (Table 1), overlapping with the sample described in previous papers from our lab [4, 5]. All participants were required to have a full-scale IQ score of 70 or above, and individuals in the ASD group met cutoff criteria for autism on both the Autism Diagnostic Observation Schedule (ADOS; [33]) and Autism Diagnostic Interview-Revised [34], administered by research-reliable examiners. Exclusion criteria for both groups included genetic disorder, neurological disorder such as epilepsy, recent history of psychiatric or learning disorders, and MRI contraindications. Children and adolescents with ASD were excluded if they were currently taking psychotropic medications, with the exception of four who were prescribed short-acting stimulant medication, but withheld it for 24 hours prior to scanning to ensure clearance [27]. No participants in the TD group had a first-degree relative with ASD, per parent report, and all were screened for behaviors consistent with an ASD diagnosis using the Social Communication Questionnaire [47]. This study was approved by the Vanderbilt University Institutional Review Board, all parents signed informed consent, and all children and adolescents signed informed assent prior to participating in study procedures.

**Table 1:** Participant characteristics

Group	ASD	TD
*N*	19	18
Age	12.58	13.11
	(2.48)	(3.44)
Full-scale IQ	109.52	104.22
	(13.96)	(12.45)
*ADOS social communication	12.32	–
	(2.79)	–
Included runs	4.58	4.50
	(0.77)	(0.71)

Demographic characteristics of participants. Values given are mean (standard deviation). * The ADOS was only administered to participants with ASD; all participants met cutoff criteria for autism spectrum on this measure (score range 9–19)

### Interest assessment

Parents were interviewed about their child’s interests and hobbies during initial phone screening. Across ASD and TD groups, children were eligible for this study if (1) they engaged with their primary interest a minimum of 1 hour per day on average and (2) the content of their interest could be represented in pictures (e.g., maps or Pokemon, but not listening to music because of the difficulty in representing the salient feature(s) visually). During the study, parents of children in both groups were administered the Yale Special Interests Interview [59] regarding the primary interest chosen from the phone screen. The YSII assesses the presence or absence of a circumscribed interest based on the intensity, duration, and degree of specialized knowledge about the topic. The interests presented pictorially to children in the experimental tasks were those confirmed with the YSII to be most salient at the time of their participation in the study. While the YSII indicated that interference as a result of interests was greater in the ASD vs. TD group, *t*(29) = 6.85, *p* < 0.001 (ASD: mean = 6.27, SD = 2.46; TD: mean = 1.00; SD = 1.79), children in both groups met inclusion criteria for strong interests on the phone screen. More detailed information about interest topics of each participant is presented in Additonal file 1: Table S1.

### Procedures

The stimuli, fMRI task, imaging acquisition protocol, post-scan memory test, and data processing and analysis steps have previously been described in Cascio et al. [4, 5] but are also detailed below.

#### Stimuli

**Fig. 1:**
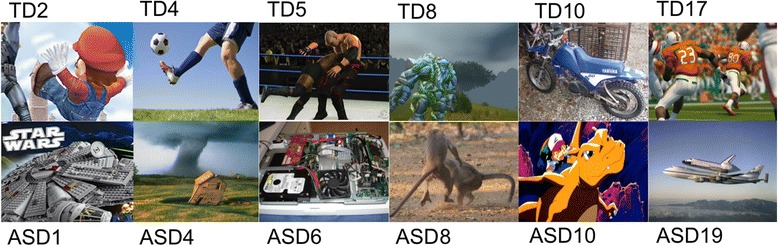
Sample stimuli from the “own interest" condition from five participants with ASD and five with TD

For this task, 38 static images from two separate conditions (76 images total) were presented. In the “own interest” condition, image sets were individually customized to depict each child’s particular area of interest (assessment of which is described in detail in [4]) . In the “others’ interest” condition, image sets consisted of a non-overlapping mix of multiple participants’ own interest images, which were the same across all participants with the exception of replacing any pictures that overlapped with the participant’s own interest. Following detailed phone screenings and parent interviews regarding each child’s interest, static images depicting this interest in landscape layout were retrieved, in .jpg format, from internet searches. Retrieved images were then manipulated in Adobe Photoshop, such that all had final dimensions of 800 × 600 pixels per inch. As imaging analyses focused on the neural response in face-processing regions, ratings were conducted to ensure that the proportion of images containing faces did not differ between ASD and TD participants. All images were independently rated regarding whether a face was present in the image (binary: yes or no) by an undergraduate research assistant, who was blind to study hypotheses and diagnostic category of the participant for which each stimulus was generated. The proportion of images containing faces within the own interest condition stimulus set was computed separately for each participant. In addition, a similar procedure was used to rate the colorfulness and visual complexity of images within the own interest condition, with individual images rated on separate 1–5 scales in terms of their colorfulness and complexity. Independent-sample *t* tests were used to determine whether the proportion of images containing faces, the mean colorfulness, or the visual complexity of images differed between the ASD and TD groups. Analyses of the content of images from individual participants’ own interest stimulus sets (Fig. 1) indicated that the ASD and TD groups did not differ in the percentage of images containing faces, *t*(35) = 1.577, *p* = .12 (ASD: mean = 65.85 %; SD = 39.94 %; TD: mean = 81.98 %; SD = 17.36 %). However, because of the significance of this potential confound, the percentage of images containing faces was used as a covariate in fMRI analyses (see below). Own interest images also did not differ between groups with regard to their colorfulness, *t*(35) = 0.036, *p* = .97, or their visual complexity, *t*(35) = 1.340, *p* = .19. Sample own interest images from a representative subset of ASD and TD participants are depicted in Fig. 1.

#### fMRI task

Before scanning, participants were acclimated to the scanner environment using a mock scanner. Participants completed a passive viewing, block design task, split across five 4-min runs. At the start of each run, instructions were presented indicating that participants should remain still and attend to each picture displayed because they would be asked about the pictures following the scan (see post-scan memory test below). Runs consisted of three 20-s blocks of each of four conditions (12 blocks total), with blocks presented in a pseudo-randomized order that was fixed across participants. Each block contained five images, each presented for 3500 ms, followed by a 500-ms presentation of a white fixation cross on a black background. In total, across all blocks and runs, participants viewed 75 images from each condition, 38 of which were unique and none of which were presented more than twice. The four conditions were as follows: (1) own interest; (2) others’ interest; (3) primary reward (not included in the current analyses of FFA response to intense interests); and (4) visual baseline. The “visual baseline” blocks consisted of images from the experimental conditions that had been rotated 180° and subjected to a Gaussian blur. Images were presented in randomized order across the five runs using Eprime 2.0 (Psychology Software Tools, Inc., Sharpsburg, PA). They were projected onto a screen behind the scanner bore that participants viewed with a mirror attached to the head coil.

#### Image acquisition

fMRI data were acquired using a 3.0 Tesla Philips Achieva MRI scanner with an eight-channel SENSE head coil. Whole-brain functional images were acquired using axial oblique slices (tilted 15° anterior higher than posterior relative to the AC-PC line) with an isotropic 2.5-mm^3^ voxel size (TR = 2 s, TE = 25 ms, flip angle = 90°, acquisition matrix = 96 × 96, no gap). The first two volumes of each functional run were discarded for equilibration. High-resolution anatomical images were acquired in the sagittal plane using a T1-weighted volumetric 3D SPGR sequence (TR = 7.9 ms, TE = 3.7 ms, flip angle = 7°, acquisition matrix: 256 × 256, 1-mm^3^ isotropic resolution). Foam cushioning between participants’ head and the birdcage coil was used to minimize motion. During the structural, scout, and reference scans, participants watched a preferred video, with the restriction that it could not be related to the topic presented in their own interest condition. During the functional scan, participants were instructed simply to pay attention to each picture. They were informed prior to scanning that they would be tested after the scan regarding how many pictures they remembered.

#### Post-scan memory test

Immediately following the scanning session, participants were tested with a computer-based task to confirm they had been attending during the passive viewing scanning paradigm. In this task, the 38 previously viewed “own” and “other” images were presented, in randomized order, along with 19 novel images from each condition. Participants were instructed to press “1” on the keyboard if they had seen the image in the scanner and “2” if they had never seen it before. Participants received feedback regarding the accuracy of their response for each trial. For each condition, hit and false alarm rates were calculated and Z-scored to compute d prime. For final analyses, fMRI data were excluded for participants whose d prime was less than 1.32 (a value corresponding to a 75 % correct rate for both old and new images). Using this criterion, imaging data from three children in the TD group were excluded. Groups did not differ on post-scan memory task performance; own interest: *t*(29) = 0.57, *p* = .57 (ASD: mean = 6.13; SD = 3.08; TD: mean = 5.50; SD = 3.10), other interest: *t*(29) = 0.73, *p* = .47 (ASD: mean = 5.24; SD = 1.91; TD: mean = 4.78; SD = 1.56).

#### Image preprocessing and quality assurance

Images were analyzed using SPM8 running in Matlab 7.13.0 (R2011b) (http://www.fil.ion.ucl.ac.uk/spm/). For each run, functional images were realigned to the first volume and resliced. All realigned functional volumes were then warped to the standard Montreal Neurological Institute (MNI) template brain, and normalized functional images were smoothed with a Gaussian kernel of 6 mm FWHM. Realignment parameters used to identify runs with motion criteria exceeding 3-mm translation and/or 3° rotation led to the exclusion of four participants (ASD: *n* = 2; TD: *n* = 2) who had fewer than three functional runs meeting inclusion criteria.

Overall, between exclusions made for poor performance on the post-scan memory test and excess motion, two participants with ASD and five TD participants were excluded, yielding a final sample of 19 in the ASD group and 18 in the TD group. Independent samples *t* tests confirmed that the final groups did not differ in age, *t*(35) = −.54, *p* = .59, IQ, *t*(35) = 1.22, *p* = .23, or mean number of included runs, *t*(35) = 0.33, *p* = .75. Final ASD and TD groups also did not differ in either average translational, *t*(35) = 0.633, *p* = .53 (ASD: mean = .0041; SD = .07; TD: mean = −.0096; SD = .06), or rotational, *t*(35) = 0.068, *p* = .95 (ASD: mean = .00065; SD = .0015; TD: mean = .00069; SD = .0015), motion for included runs. Characteristics of the final sample are reported in Table 1.

#### Statistical analysis

Individual-level analyses were performed using the general linear model design matrix, modeled using the canonical hemodynamic response function (HRF). To minimize the contribution of volumes with motion spikes to the overall model, the robust weighted least squares (rWLS; [16]) toolbox was used to inversely weight volumes according to their variance due to noise. Next, each model was estimated with the classical restricted maximum likelihood approach for spatially smoothed images and individual-level contrasts were created by subtracting the BOLD pattern of activation for others’ interest condition from the own interest condition (own-other), across all included runs.

Group-level analyses were completed in two stages. After specifying the general linear model with percent of images containing faces (see above) as a covariate of no interest, one-sample *t* tests were used to create within-group contrasts between conditions. Next, independent-sample *t* tests were conducted to compare contrasts between the ASD and TD groups. For all analyses, proportion of images containing faces was used as a covariate of no interest to control for differences in activation that might be confounded by differences in the face content of images.

#### Regions of interest

In an effort to improve statistical power in the absence of independent data for individual-level functional localization of FFAs, we employed an alternative method for conducting region of interest (ROI) analyses in two stages. First, for preliminary analyses, we created a broad ROI mask for the entire fusiform gyrus using automated anatomical labeling (AAL) regions from the Wake Forest University Pick Atlas [36]. This mask was applied for all group results and a threshold of *Z* >1.65 (uncorrected *p* < 0.05) with a cluster size of at least ten voxels was used to identify voxels with a statistically significant BOLD response. Significant clusters were then submitted to correction for family-wise error using AlphaSim (http://afni.nimh.nih.gov/pub/dist/doc/manual/AlphaSim.pdf). AlphaSim implements a Monte Carlo simulation procedure in order to determine the probability of a false positive from the frequency count of cluster sizes [18]. Masks for each ROI were then registered to functional image space and used with a corrected *p* value of .05 and smoothing kernel of 6 mm in 5000 iterations of the simulation procedure.

Second, in a more spatially constrained analysis, we created four 5-mm radius spherical ROI masks corresponding to bilateral posterior and anterior portions of FFA: FFA1 and FFA2, respectively [37, 46, 66]. To test for specificity of group effects to the FFA, which is part of the ventral visual stream, we created bilateral approximately 12-mm radius spheres in an extrastriate region of comparable hierarchy in the dorsal visual stream, area MT/V5. FFA masks were centered on the group-averaged peaks of face-selectivity reported by Pinsk and colleagues [46], transformed to the MNI coordinate system: rFFA1 (34, −65, −16); rFFA2 (36, −53, −19); lFFA1 (−38, −65, −18); lFFA2 (−38, −51, −18). MT/V5 masks were centered on coordinates reported by Watson and colleagues [65] transformed to MNI space: rMT (40, −67, −3), lMT (−42, −79, −3). These masks were created using Marsbar [3] and applied in all group analyses. Statistical effects were probed from within the restricted cortices. To identify clusters with statistically significant BOLD response within the masks, we used a threshold of *Z* > 1.65 (uncorrected *p* value of .05) but did not include a cluster size threshold because of the much smaller size of these ROIs relative to the broad fusiform gyrus mask.

## Results

### Within-group contrasts

**Fig. 2:**
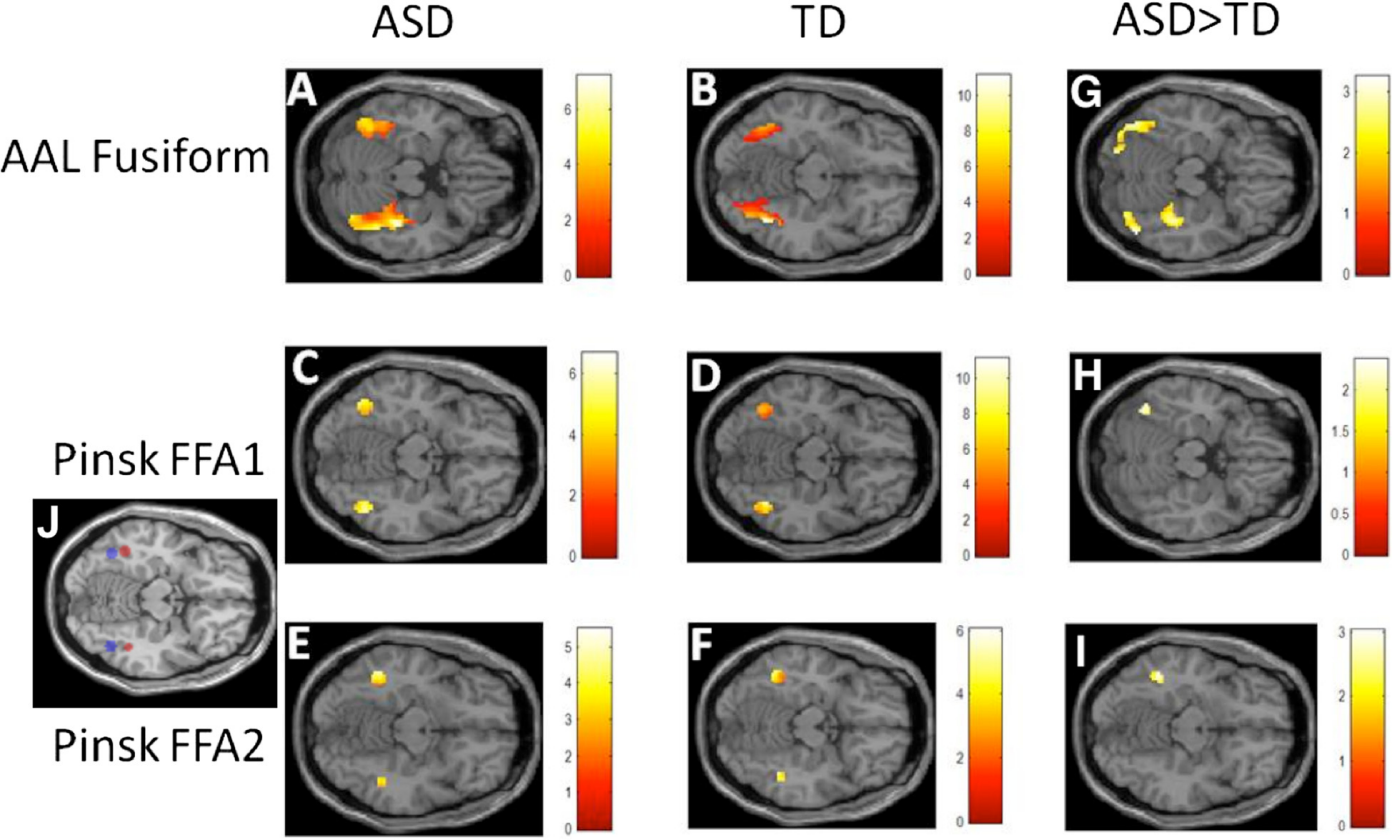
Within-group activations for the own-other contrast within the bilateral AAL-defined fusiform gyrus mask (**a**, **b**), as well as posterior FFA1 (**c**, **d**) and anterior FFA2 (**e**, **f**) masks, defined by coordinates provided in [46], show that both children with ASD and TD activated bilateral fusiform regions when viewing images of their own interests. Between-group activations within bilateral AAL-defined fusiform gyrus mask (**g**), as well as left posterior FFA1 anterior FFA2 masks (**h**, **i**), reveal that children with ASD show more robust recruitment of fusiform regions than do children with TD when viewing images of their own interest. Bilateral FFA1 (in *blue*) and FFA2 (in *red*) masks are depicted in Fig. 2 **j**

In both groups, viewing images of one’s own interest elicited significantly higher BOLD signal relative to viewing images of other children’s interest (own-other) in discrete clusters within the FFA masks. These findings held both within the broad AAL-defined fusiform gyrus mask (Table 2, Fig. 2a, b) as well as within the more precise bilateral FFA1 and FFA2 masks (Table 3, Fig. 2c–f).

**Table 2:** Within-group contrasts for AAL-defined fusiform gyrus masks

Own-Other							
Group	Region	*x*	*y*	*z*	*K*	*Z*_max_	*p*_(uncorr)_
ASD	Right FG mask	40	−33	−20	834	4.82	.000
	Left FG mask	−45	−63	−18	550	4.08	.000
TD	Right FG mask	40	−58	−13	480	5.81	.000
		30	−5	−30	51	3.03	.001
	Left FG mask	−45	−58	−20	410	4.29	.000
		−40	−20	−28	16	3.02	.001

Significant clusters within AAL-defined fusiform gyrus (FG) mask that survived AlphaSim correction for multiple comparisons for ASD and TD groups separately. Significance threshold of *Z* > 1.65 (uncorrected *p* < 0.05) with a cluster size of at least ten voxels was applied. Coordinates are given in MNI space and represent the peak of the activation cluster. *K* = number of 2.5-mm^3^ voxels; *Z* = height threshold; *p* = uncorrected significance level

**Table 3:** Within-group contrasts for Pinsk ROIs

Own-Other							
Group	Region	*x*	*y*	*z*	*K*	*Z*_max_	*p*_(uncorr)_
ASD	R FFA1 mask	45	−58	−13	32	4.51	.000
	R FFA2 mask	43	−48	−20	23	4.11	.000
	L FFA1 mask	−40	−65	−13	41	4.01	.000
	L FFA2 mask	−45	−48	−13	42	3.52	.000
TD	R FFA1 mask	40	−60	−13	32	5.64	.000
	R FFA2 mask	43	−48	−18	23	4.30	.000
	L FFA1 mask	−40	−63	−15	41	4.24	.000
	L FFA2 mask	−45	−50	−20	42	3.93	.000

Significant clusters within 5-mm-radius spherical ROIs defined from [46]. A significance threshold of *Z* > 1.65 (uncorrected *p* < 0.05) was applied. Coordinates are given in MNI space and represent the peak of the activation cluster. *K* = number of 2.5-mm^3^ voxels; *Z* = height threshold; *p* = uncorrected significance level

### Between-group contrasts

When directly comparing the ASD and TD groups, the “own-other” contrast elicited significantly greater response in ASD than TD in both left and right fusiform regions (Table 4, Fig. 2g). In contrast, no clusters within the fusiform gyrus showed greater BOLD response to the own-other contrast in the TD group, relative to the ASD group. In the more stringent analyses using separate ROI masks defined from coordinates Pinsk et al. [46] identified for left and right FFA1 and FFA2, the own-other contrast again elicited greater response in the ASD than the TD group. Specifically, there were clusters within both left and right FFA1 and FFA2 masks that showed significantly greater BOLD signal in ASD than TD (Table 5, Fig. 2h, i). Similar to analyses of the broader fusiform gyrus, there were no clusters in either hemisphere that elicited greater response in the TD relative to the ASD group for own-other contrast within the FFA1 or FFA2 masks. There were no significant group differences in either direction for the control extrastriate region MT/V5.

**Table 4:** Between-group contrasts for AAL-defined fusiform gyrus mask

Own-Other							
Group	Region	*x*	*y*	*z*	*k*	*Z*_max_	*p*_(uncorr)_
ASD > TD	Right FG mask	25	−80	−18	113	3.09	.001
		20	−33	−23	173	3.06	.001
	Left FG mask	−45	−73	−18	185	3.12	.001
TD > ASD	[None]						

Significant clusters within AAL-defined fusiform gyrus (FG) mask for between group comparisons of the own-other contrast that survived AlphaSim correction for multiple comparisons. Significance threshold of *Z* > 1.65 (uncorrected *p* < 0.05) with a cluster size of at least ten voxels was applied. Coordinates are given in MNI space and represent the peak of the activation cluster. *K* = number of 2.5-mm^3^ voxels; *Z* = height threshold; *p* = uncorrected significance level

**Table 5:** Between-group contrasts for Pinsk ROIs

Own-Other							
Group	Region	*x*	*y*	*z*	*k*	*Z*_max_	*p*_(uncorr)_
ASD > TD	R FFA1 mask	48	−63	−15	1	1.70	.044
	R FFA2 mask	43	−43	−15	1	1.70	.044
	L FFA1 mask	−40	−60	−18	5	2.04	.021
	L FFA2 mask	−43	−45	−13	24	2.81	.002
TD > ASD	[none]						

Significant clusters within 5-mm-radius spherical ROIs defined from [46]. A significance threshold of *Z* > 1.65 (uncorrected *p* < 0.05) was applied. Coordinates are given in MNI space and represent the peak of the activation cluster. *K* = number of 2.5-mm^3^ voxels; *Z* = height threshold; *p* = uncorrected significance level

Two three-way interactions were conducted to evaluate whether group differences in FFA activation to objects of ones own interest were affected by laterality (right vs. left) or location (anterior vs. posterior) of the FFA region for either cluster size or height threshold. Results of the analyses for cluster size revealed a significant main effect of location, *F*(1,35) = 17.92, *p* < 0.001, and a significant laterality by location interaction, *F*(1,35) = 22.70, *p* < 0.001, but no interactions including group. Results of the analyses for height threshold revealed a significant main effect of location, *F*(1,35) = 10.23, *p* = 0.003, but no other main effects or interactions. Pairwise post hoc tests to explore the nature of significant effects revealed that, across ASD and TD groups, the extent of activation was significantly more robust in FFA1 versus FFA2 in the right hemisphere, *t*(36) = 6.190, *p* < 0.001, whereas FFA1 and FFA2 activation clusters in the left hemisphere were highly similar, *t*(36) = 0.015, *p* = 0.99.

## Discussion

In the current study, we explored whether the fusiform gyrus, a hub for expert level individuation of faces and objects, was responsive to customized stimulus sets reflecting the time-intensive hobbies and interests of children and adolescents with and without ASD. Our results indicate that images from topics of interest can elicit response from expertise-responsive regions in both ASD and TD individuals, but that this response is more robust in ASD. This finding is consistent with the notion that the intensity of restricted interests in ASD [62] is likely to result in unusually high levels of perceptual expertise. Although we did not ask participants to perform a perceptual discrimination task among exemplars in the categories of their topic of interest, the enhanced recruitment of the FFA in the ASD group provides indirect support for this intuitive idea.

Visual expertise has been associated with increased reward system activation, particularly in the medial orbital frontal cortex (OFC) and cingulate Kirk et al. [28]. We have previously shown that both the amygdala and the OFC are engaged by individuals with ASD when viewing images of their restricted interest, with the OFC being particularly selective for images of one’s own interest versus novel pictures of others’ interest [4]. This finding demonstrated that (1) areas of the brain that subserve social functions are not globally impaired or under-responsive in ASD, and (2) reward processing regions may be especially responsive to circumscribed areas of interest in ASD, which often involving objects and other non-social stimuli. These results converge with the current findings to suggest that both reward and expertise-related visual processing areas show enhanced response to topics of intense interest in ASD. These findings could offer insight into mechanisms by which neural networks involved in social behavior could develop atypical specialization in ASD.

Johnson [23] proposed that, early in development, subcortical limbic structures including the amygdala ensure infants attend preferentially to faces, which initially provides extensive visual input to specific areas in the fusiform gyrus, and ultimately leads to the development of a specialized face-processing region, or the FFA [23, 24]. If limbic structures such as the amygdala are less responsive to human faces in ASD and more responsive to objects or scenes related to restricted interests throughout development, this mechanism could lead to atypical cortical specialization of traditionally face-responsive regions. Thus, as we show here, regions of the brain that are typically responsive to social stimuli (faces) may function better in their other capacities; in this case, the FFA in its role as a hub for perceptual expertise. Investigation of limbic and reward circuitry in infants who will later develop ASD is an important area for further study in order to determine the developmental course by which atypical neural specialization within social brain regions emerges in ASD.

Although some percentage of both groups’ topics of interests in this study included images that *contained* faces (e.g., several football players engaging in a tackle, cartoon scenes with landscape and entire figures), none of them had faces as a sole or primary focus (as stimuli that optimally recruit the FFA do). Our finding of greater activation to own vs. other images in *both* ASD and TD provide support, across both typical and atypical development, in favor of the FFA as a broader expertise region, rather than a face-specific region. Although there was not a significant group difference in the number of images that contained faces, there was a trend for that number to be higher in the TD group. We accounted for this by covarying for the percentage of images that contained faces within each participant’s own image set. Importantly, if the lower representation of faces in one group’s stimulus set were to have an effect on FFA activation, it would be expected to result in reduced FFA activation relative to the second group, whose images contained more faces. However, our results are in the opposite direction; we saw enhanced, not diminished, response of FFA in the ASD group, whose image sets contained marginally fewer faces. Future studies directly comparing FFA response to both faces and topics of special interest in a carefully selected population of individuals with and without ASD whose interests do not relate to topics where faces may be present would be helpful for further parsing the question of specialization in the FFA in ASD.

Our findings are also consistent with the idea that diminished responses of regions that are typically interrogated with face stimuli may not reflect generalized pathology in ASD but rather differential tuning [60] toward stimuli that are less conventionally social than isolated faces, such as vehicles or anime scenes. While the literature reporting reduced FFA response to faces in ASD is somewhat mixed, it does seem clear that human faces do not engage this region to the same extent and with the same reliability as in TD individuals [8, 56, 57], particularly in individuals with severe symptoms [52]. It is unknown at what point in development this difference may emerge, but early failure to develop specialized function within the neuroanatomical structures typically subserving social functions may recursively shape the response properties of these structures, ultimately leading to difficulty engaging in the social world for people with ASD [43]. In infants later diagnosed with ASD, decreased visual attention to social scenes is evident by six months of age [6], while in the same developmental window attention to non-social objects is enhanced [17]. This preference for looking at objects is sustained throughout early childhood [48, 49, 51]. Thus, differential visual salience of environmental (versus conventionally social) stimuli may result in atypical focus on these stimuli, leading to differently appropriated visual expertise, and in turn predisposing the FFA to respond preferentially to these stimuli in ASD.

The localization of posterior and middle portions of the FFA is still a relatively new convention in the literature [46, 66]. Little is known about the response property distinctions or similarities in these posterior and anterior portions; however, a recent study with typical subjects showed evidence that FFA1 and FFA2 both respond to non-face objects of expertise but that bilateral FFA2 may be most robust to conditions of competition [38]. In addition, neuroanatomical work has shown that cross-correlations in cortical thickness of within-hemisphere FFAs (rFFA1 × rFFA2 and lFFA1 × lFFA2) are higher in the left hemisphere than the right (McGugin, Van Gulick, Gauthier, submitted). Consistent with this work, our results indicated that the extent of the own-other response for both TD and ASD populations was significantly different for anterior and posterior FFAs in the right hemisphere, with no difference in the response properties of FFAs in the left hemisphere. These data support the hypothesis that the two right FFAs may be more distinct relative to the two left FFAs. In addition, they support the idea that, for objects of expertise, the FFA functions similarly in ASD as in TD. However, caution should be taken when interpreting these results, given that in the current work, posterior and middle portions of the FFA were not localized on an individual subject basis, so it is possible that they reflect parts of a single FFA.

Our findings have important translational implications. Recent research has demonstrated that responsivity of regions of the social brain can be modulated by behavioral intervention targeting social motivation in children with ASD [63]. Specifically, children with ASD who initially showed atypical recruitment of posterior STS during a social perception task exhibited increased compensatory activation in related brain regions, including the amygdala and striatal reward regions, following administration of pivotal response treatment (PRT). These neural changes paralleled behavioral and clinical evidence showing improvement in social functioning in treated children [64]. The fact that both the reward system and FFA can be robustly recruited in ASD given stimuli of appropriate salience provides an additional avenue for intervention. For example, pairing stimuli of particular interest and expertise (which more automatically engage reward and visual expertise regions in ASD that are also robustly recruited by social stimuli in TD) with social input or interactions (e.g., a smile, eye contact, a verbal request directed to another person) may serve to shape these regions toward responding more robustly to social input in ASD as well. Thus, it is possible that PRT and other interventions targeting social motivation may be able to modify and normalize responsiveness of the FFA for faces, harnessing topics of interest and expertise to increase neural specialization for social stimuli and thereby perhaps offering a mechanism by which the brain may enable more effective functioning within the social world. This possibility could be explored directly through intervention studies where neural response in “social” brain regions, including FFA and reward systems, is probed with stimuli reflecting both social and restricted interest categories in order to test the degree to which these regions interact and are shaped both over the course of development and through behavioral pairings during intervention.

Our study utilized a unique design featuring personalized stimuli to assess recruitment of a social brain region during viewing of images relates to individuals’ topics of intense interest. The use of individualized stimulus sets enabled us to explore recruitment of expertise-responsive brain regions to participants’ specific hobbies and interests. However, this design inherently introduced heterogeneity of stimuli across individuals. We addressed this issue in several ways. First, we used a standard size and resolution for each image. Second, analyses indicated that neither visual complexity nor proportion of images containing faces differed between groups. Third, we statistically controlled for individual differences in the degree to which faces were present in an individual’s stimulus set by adding a covariate to the model. Another possible limitation of this study is that we used a passive viewing task and did not use eye tracking or measure dwell time while participants viewed task images; however, participants were asked to view and remember presented images for post-scan testing, an exclusion criterion was used based on post-scan memory test performance, and groups did not differ in their performance on this task. In this study, the FFA region was not localized on an individual subject basis using a canonical face localizer task; however, the appropriateness of using such a localizer task in a clinical population with known face-processing impairments could be questionable. Despite the lack of localizer, both broader analyses of the entire fusiform gyrus and more stringent analyses limited to literature-based coordinates for anterior and posterior FFA regions converged in showing that individuals with ASD recruited fusiform clusters more robustly than individuals with TD when viewing images of their interest. Finally, though our aims focused on analyses of group differences in the FFA and we controlled for nonspecific extrastriate group differences by examining area MT/V5, it is possible that results we observe are reflective of broader differences within the ventral visual stream.

## Conclusions

This study used a novel task design to probe whether the fusiform gyrus can be effectively recruited by individuals with ASD in the context of viewing images of their topics of intense interest. Whereas studies using face stimuli have often shown decreased recruitment of the FFA in ASD, our results reveal that this region is recruited to an enhanced degree in ASD for objects of intense interest, which are likely highly familiar and thus associated with perceptual expertise. This finding is consistent with a previously reported case study [20] and with previous work indicating intact FFA response to familiar faces in ASD [45]. Taken together, these findings support the notion that key nodes of the social brain are not globally dysfunctional in ASD, but rather may be differentially tuned toward responding to stimuli of particular familiarity, interest, or expertise, which are typically less conventionally social than stimuli of unfamiliar faces used in most research studies. Future studies could examine FFA response to both face and expertise-related stimuli concurrently and could limit expertise-related stimuli to objects or scenes with no human figures to determine more directly the extent to which increased FFA recruitment for processing non-social stimuli may come at the expense of its recruitment for social stimuli. This line of work may prove informative both for understanding the neural basis of restricted interests in ASD and for developing and refining interventions targeting development of expertise, efficiency, and proficiency with processing social information.

## Additional file

Additional file 1: Table S1. Interest topics for ASD and TD participants. (DOC 36 kb)
