# Supplementary material for: A functional neuroimaging study of fusiform response to restricted interests in children and adolescents with autism spectrum disorder
Source: J Neurodev Disord. 2016 Apr 14;8:15. doi: 10.1186/s11689-016-9149-6 (PMC4831124; doi:10.1186/s11689-016-9149-6)
Supplement: Additional file 1: Table S1. — Interest topics for ASD and TD participants. (DOC 36 kb) [file 11689_2016_9149_MOESM1_ESM.doc]

Table S1. Interest topics for ASD and TD participants.

| ASD1 | Bionicles/Lego star wars | TD1 | Dirtbikes |
| --- | --- | --- | --- |
| ASD2 | Maps | TD2 | Pokemon and Mario |
| ASD3 | Hollywood/Film Make-up | TD3 | NFL video game |
| ASD4 | Natural Disasters | TD4 | Soccer |
| ASD5 | Instruments and record players | TD5 | WWE wrestling video game |
| ASD6 | Computer technology | TD6 | Baseball |
| ASD7 | Mario and Zelda | TD7 | Trumpet/ Band |
| ASD8 | Primates | TD8 | World of Warcraft |
| ASD9 | Sonic, Mario, and Zelda | TD9 | NHL & NFL |
| ASD10 | Pokemon | TD10 | Pokemon |
| ASD11 | Pokemon | TD11 | Dirtbikes/ Skillet |
| ASD12 | Bionicles/ Kingdom Hearts | TD12 | Xbox NCAA football games |
| ASD13 | Deadliest Warrior / Ninja Warrior | TD13 | Basketball |
| ASD14 | World of Warcraft | TD14 | Titans/Lakers |
| ASD15 | Zelda / Pokemon | TD15 | Wrestling, Eagles, and Predators |
| ASD16 | The Simpsons | TD16 | UT Basketball and Titans Football |
| ASD17 | Pokemon/Lego Star wars | TD17 | Baseball (Texas Rangers), NCAA football games on Xbox |
| ASD18 | Anime | TD18 | Skillet |
| ASD19 | Airplanes |  |  |
